# Acupuncture-related techniques for postoperative cognitive complications: a systemic review and meta-analysis

**DOI:** 10.1186/s13741-023-00303-5

**Published:** 2023-05-03

**Authors:** Junbao Zhang, Zhuoma Cairen, Liwen Shi, Minjuan Zhang, Manping Yang, Yun Wang, Zhihong Lu

**Affiliations:** 1 Department of Anesthesiology, Xijing Hospital, Fourth Military Medical University, Xi’an, 710032 Shaanxi China; 2 Department of Anesthesiology, Qinghai Provincial People’s Hospital, Xining, 810007 Qinghai China

**Keywords:** Acupuncture, Postoperative cognitive dysfunction, Meta-analysis

## Abstract

**Background:**

Postoperative cognitive complications are major challenges for postoperative recovery. Acupuncture-related techniques have been used for treating neurocognitive dysfunctions. However, whether they help to prevent postoperative cognitive complicationss remains unclear. We intend to evaluate the effect of acupuncture-related techniques on the incidence of postoperative cognitive complications (PCC) in patients undergoing surgery under general anesthesia.

**Methods:**

Based on PRISMA guidelines, a search of PubMed, EMBASE, Web of Science, and the Cochrane Central Register of Controlled Trials and ClinicalTrials.gov was performed to identify eligible trials published from inception to June 6, 2021. The search was performed in June 2021. The inclusion criteria were prospective, randomized, controlled clinical trials that compared acupuncture-related techniques with other techniques or non-acupuncture treatment in patients undergoing surgery under general anesthesia. Pooled odds ratios (ORs), 95% CIs, and* P* values were estimated for end points using fixed and random effects statistical models.

**Results:**

The analysis included 12 studies with 1058 patients. Compared with patients not receiving acupuncture, patients treated with acupuncture-related techniques had a lower incidence of PCCs (OR, 0.44; 95% CI, 0.33 to 0.59; *P* < 0.001; *n* = 968) and lower levels of biomarkers, including IL-6, TNF-α, and S100β. Acupuncture with needles and without needles showed similar effects on the prevention of PCCs. The effects of acupuncture-related techniques on PCCs were observed in both English and non-English articles. Subgroup analyses showed that both agitation and/or delirium (OR, 0.51; 95% CI, 0.34 to 0.76; *P* < 0.001; *n* = 490) and delayed cognitive recovery (OR, 0.33; 95% CI, 0.21 to 0.51; *P* < 0.001; *n* = 478) were reduced after treatment with acupuncture-related techniques. In adult studies evaluating MMSE scores, the scores were not different between groups (SMD, − 0.71; 95% CI, − 1.72 to 0.3; *P* = 0.17; *n* = 441).

**Conclusions:**

Acupuncture-related techniques, including needle techniques and electrical techniques, are associated with fewer postoperative cognitive complications, suggesting that acupuncture could be considered a potential option in the perioperative setting. Additional research is needed to develop higher-quality evidence and optimal regimens.

**Trial registration:**

PROSPERO (CRD42021258378).

**Supplementary Information:**

The online version contains supplementary material available at 10.1186/s13741-023-00303-5.

## Background

Postoperative cognitive complications consisting of agitation and/or delirium and long-lasting postoperative cognitive dysfunction are great challenges for anesthesiologists. Patients, especially those in the aging population, who suffer from postoperative cognitive complications could be at higher risk of morbidity and mortality (Olotu 2020). Growing evidence suggests a possible role for neuroinflammation, reduced functional connectivity, reduced glucose utilisation, and neurotransmitter imbalances, particularly involving dopamine and acetylcholine, in the processes underlying postoperative cognitive complications (PCCs) (Cibelli et al. 2010; Hu et al. 2018; Subramaniyan and Terrando 2019; Dellen et al. 2014; Vasunilashorn et al. 2015).

Management of postoperative cognitive complications involves a multi-professional approach and consists of pharmacological and nonpharmacological components (Olotu 2020). No single medication or intervention to prevent or treat postoperative cognitive complications is available. Avoiding anesthesia that is too deep, avoiding large swings in hemodynamics, effective pain management, and early mobilization are reported to be of benefit (Guenther et al. 2016; Heinrich et al. 2021; Li et al. 2020a; Zuylen et al. 2021).

Acupuncture-related techniques have been used for the treatment of cognitive disorders such as dementia after stroke and mild cognitive impairment (Du et al. 2020; Li et al. 2019, 2020b; Min and Xu-Feng 2016; Wang et al. 2016; Yang et al. 2016). Furthermore, the role of acupuncture in the perioperative scenario are reported in both animal and clinical studies (Lu et al. 2015; Ho et al. 2020). Possible mechanisms include acupuncture modulating inflammation, oxidative stress, synaptic changes, and other cellular events to mitigate cognitive disorders (Mazidi et al. 2017; Liu et al. 2017; Yuan et al. 2014; Silva et al. 2015; Yang et al. 2018). Perioperative acupuncture reduces not only the consumption of anesthetics and analgesics but also anesthesia-related side effects (Yang et al. 2016). Subsequent studies have assessed the effects of acupuncture-related techniques on postoperative cognitive complications, but there has not been a pooling of their findings (Ho et al. 2020). Therefore, we performed a systematic review and meta-analysis of the efficacy of acupuncture-related techniques to prevent postoperative cognitive complications in patients undergoing general anesthesia. Our primary outcome was the incidence of postoperative cognitive complications, and we reported the severity of postoperative cognitive complications and changes in preinflammatory cytokines.

## Material and methods

We adhered to the guidelines of the Preferred Reporting Items for Systematic Reviews and Meta-Analyses (PRISMA) statement for the conduct and reporting of this systematic review. The protocol was registered at PROSPERO (CRD42021258378).

A systematic search with no restriction to language and publication status was performed on June 4, 2021. We searched PubMed, EMBASE, Web of Science, the Cochrane Central Register of Controlled Trials and ClinicalTrials.gov for eligible studies. We reviewed the reference lists of the included publications and previous systematic reviews to identify additional eligible studies. The search strategy is detailed in Additional file 1: Supplemental file 1 (eMethod). We did not search the gray literature.

The inclusion criteria were prospective, randomized, controlled clinical trials that compared acupuncture-related techniques with other techniques or non-acupuncture treatment for postoperative cognitive complications in patients undergoing surgery under general anesthesia. We excluded observational studies and quasi-randomized and nonrandomized controlled trials. We excluded studies without assessment of postoperative cognition. This meta-analysis of readily available literature did not require institutional review board approval, and each respective study detailed their consent procedures.

Our primary outcomes included the incidence of postoperative cognitive complications during the hospital stay. Postoperative cognitive complications include emergence agitation and/or delirium, postoperative cognitive dysfunction, and postoperative delirium. When postoperative cognitive complications were assessed several times after surgery, the highest incidence of a postoperative cognitive complications during the hospital stay was extracted. Our secondary outcomes included the highest mini-mental state examination (MMSE) scores during the hospital stay. and levels of inflammatory cytokines and cerebral injury biomarkers, including interleukin (IL-6), tumor necrosis factor (TNF)-α, and S100β.

Data extraction was independently performed by two authors (M.Z. and J.Z.), with good interobserver agreement (κ = 0.95). These two authors independently screened the articles and extracted the following data from each study: patient characteristics (age, sex, and American Society of Anesthesiologists [ASA] physical status), study characteristics (country, type of surgery and anesthesia, sample size), intervention characteristics (type of acupuncture, timing and comparators), and outcomes of interest (assessment tool, type of PCC).

Z.L. and L.S. independently evaluated the risk of bias for each study included with the Cochrane risk of bias assessment tool (Higgins and Green 2008). We also reviewed conflicts of interest or industry sponsorship. We resolved any inconsistency through discussion (κ = 0.55–1.0). When an email address was available, we contacted the authors of the original study for detailed information. Specifically, we attempted to retrieve information regarding the detailed methodology of each trial and the outcomes of interest that were not presented in the articles. We deemed the authors to be unresponsive if they did not reply after three consecutive attempts, based on a previous study (Kuriyama and Maeda 2019). The overall certainty of evidence for each outcome was assessed using the Grading of Recommendations Assessment, Development and Evaluation (GRADE) approach (Balshem et al. 2011).

We calculated the odds ratios (ORs) and standardized mean differences (SMDs) for dichotomous and continuous outcomes, respectively. Pooled ORs and *P* values were estimated for the incidence of postoperative cognitive complications using the Mantel–Haenszel method and either a fixed or random effects statistical model. When a study presented the data as the median with interquartile range, we converted the values to the mean and standard deviation. Meta-analyses were conducted with Review Manager software (RevMan; version 5.1 for Windows; Nordic Cochrane Centre). The 95% CIs were calculated and are presented in forest plots. Statistical heterogeneity was evaluated with the *χ*^2^ test, and inconsistency was estimated using the I^2^ statistic. We conducted subgroup analysis based on patient population, type of acupuncture, and type of postoperative cognitive complications. The quality of the selected randomized clinical trials was assessed based on the instrument developed by Jadad et al. (Jadad et al. 1996; Spring et al. 2016) (Additional file 1: Table S1). Sensitivity analyses were conducted to evaluate the effect of acupuncture on postoperative cognitive complications in studies published in English and non-English and in studies using different anesthetic techniques. When a trial had zero events in either arm, we performed sensitivity analyses with continuity corrections by adding 1 to each cell of the 2 × 2 tables from the trial (Kuriyama and Maeda 2019; Sweeting et al. 2004). *P* values of 0.05 were considered statistically significant.

Egger’s regression test was used to assess publication bias for the primary outcomes of this review. We also created a funnel plot for the assessment of publication bias in situations of low risk of publication bias.

## Results

### Overview of included studies

**Fig. 1:**
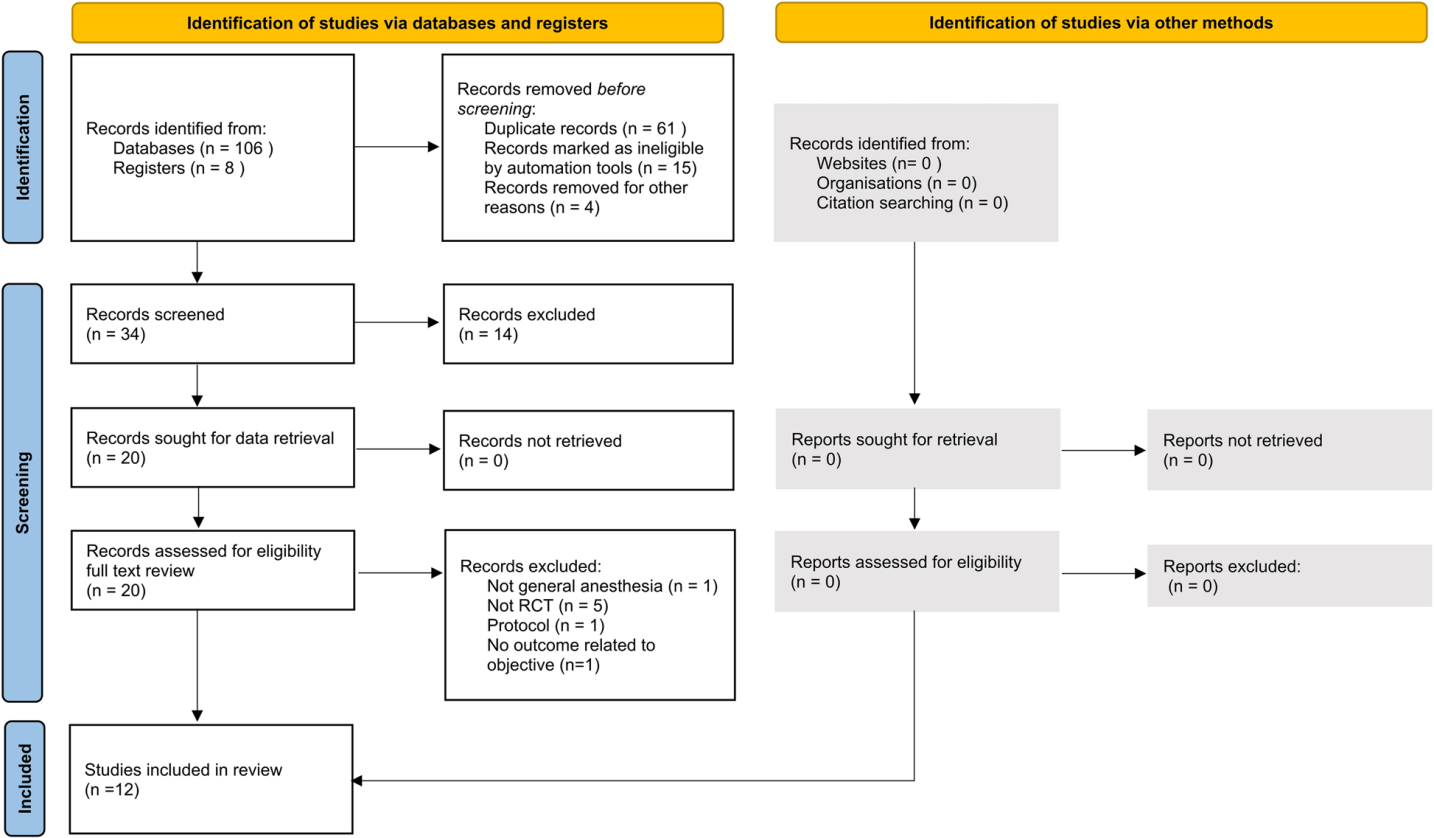
PRISMA flow diagram for the systemic review

Our database search initially produced 106 titles and abstracts. Thirty-four records were finally screened. Twenty-one non-RCT records were excluded. One record was excluded due to lack of assessment of postoperative cognition. We ultimately included 12 randomized controlled trials involving 1058 study participants for the analysis after applying the inclusion and exclusion criteria (Fig. 1). The risk of bias for each study was shown in Additional file 1: Fig. S1.

Eight and four trials included patients older than 65 years old and younger than 10 years old (1.5–8 years old for two trials, 2–10 years for one trial, 1–6 years for one trial), respectively. Eight trials were conducted in China. The types of surgery were listed as follows: ENT surgery (adenoidectomy and/or tonsillectomy, myringotomy tube placement), gastrointestinal surgery, interventional surgery, and orthopedic surgery (spine, knee replacement) (Table 1).

**Table 1:** Summary of the findings of the clinical trials included in the meta-analysis

Study	Age	Country	Sample size (female)	ASA	Type of Surgery	Anesthesia methods	Type of acupuncture	Paramenters for stimulation	Acupoints	Comparator	Timing of acupuncture	Type of PCC	Time of PCC	Assessment tool	Primary outcome	Other outcomes
Acar et al. (2012)	2-10yrs	Turkey	50(25)	I-II	Adenoidectomy and/ or tonsillectomy	Sevo	capsicum plasters	NA	bilateral HT7	inactive plasters	30 min before anesthesia induction	agitation	15 min after surgery	PAED scale	Incidence of agitation	Pain
Gao et al. (2012)	> 65yrs	China	120(68)	I-III	Non-cardiac surgery	Sevo	TEAS + electroacupuncture	2/100 Hz	Hegu, Neiguan, Zusanli, Baihui	no acupuncture	30 min before anesthesia induction to end of surgery	POCD	2,4,6 d after surgery	MMSE	Incidence of POCD	PONV, MMSE scores
Lin et al. (2013)	≥ 65yrs	China	124 (42)	II-III	Gastrointestinal surgery	Propofol	TEAS	2/100 Hz	Baihui, Yintang, Neiguan	no acupuncture	30 min before induction to end of surgery	POCD	3d post-op	MMSE	Incidence of POCD	S100β
Lin et al. (2014)	≥ 65yrs	China	83(29)	I-II	Gastrointestinal surgery	Propofol	electroacupuncture	2/100HZ	Neiguan, Zusanli, Baihui	no acupuncture	30 min before anesthesia induction to end of surgery	POCD	3d after surgery	MMSE	Incidence of POCD	Time to awake, Remifentanil consumption,TNF-A, IL-1B, IL-6
Hijikata et al. (2016)	18-96 months	Japan	120(37)	I-II	Minor surgery	Sevo	TEAS	1 Hz	HT7	Sham (electrode without stimulation)	during surgery	emergence agitation	during PACU	PAED, Aono's scale	Incidence of agitation	Time to tracheal extubation, PACU stay duration and postoperative pain scores
Yuan et al. (2016)	29-72yrs	China	122(ND)	I-III	Intervention neurosurgery	Propofol	TEAS	1.5 Hz	Baihui, Yintang, Neiguan	no acupuncture	before anesthesia induction	POCD	post-op 7ds,30ds	MMSE	Incidence of POCD	NSE 、S-100β、IL-1β、IL-6 TNF-α
Zhang et al. (2017)	> 65yrs	China	90(42)	I-II	Spine surgery	Propofol	electroacupuncture	2/15 Hz	Baihui, Dazhui, Zusanli	no acupuncture	30 min before induction to end of surgery	POCD	post-op 3ds	MMSE	Incidence of POCD	Interleukin (IL)-6, IL-10, and S100b level; remifentanil, propofol consumption
Gao et al. (2018)	> 65yrs	China	64(31)	I-II	Spine surgery	Propofol	TEAS	2/100 Hz	Hegu, Neiguan	Sham (electrode without stimulation)	30 min before induction to end of surgery	POD	post-op 3ds	RASS,CAM	Incidence of POCD	TNF-α, IL-6, matrix MMP-9, and S100β level; remifentanil, propofol consumption
Nakamura et al. (2018)	18-96 months	Japan	100(26)	I-II	Inguinal hernia repair or orchiopexy	Sevo	TEAS	1 Hz	HT7	Sham (electrode without stimulation)	during surgery	emergence delirium	In PACU	PAED, Aono's scale	Incidence of agitation	The severity of EA, PACU stay duration, and postoperative pain
Zhao et al. (2018)	65-75yrs	China	60(34)	Not descripted	Knee replacement	Propofol	electroacupuncture	2/100 Hz	Tou Sanshen, Baihui, Hegu, Taichong	placebo needle	5 days before sugery, once daily	POCD	post-op 1d, 7d	MMSE	MMSE score	TNF-α, IL-6, and S100β
Martin et al. (2020)	1-6yrs	USA	99(38)	I-III	Myringotomy tube placement	Sevo	acupuncture	NA	HT7, ear Shenmen	no acupuncture	during surgery	emergence delirium	in PACU	PAED	Highest PaeD score	Post-discharge agitation and sleep quality
Liu et al. (2021)	≥ 65yrs	China	100(49)	I-II	Radical colon rectomy	Propofol	TEAS	2/100 Hz	Hegu, Neiguan, Zusanli	Sham (electrode without stimulation)	30 min before induction to end of surgery	POCD	1d,3d post-op	MMSE	Incidence of POCD	IL-6, hs-CRP and CGRP Levels

*RCT* Randomized clinical trial, *TEAS* Transcutaneous electrical acupoint stimulation, *PAE* Post-anesthetic emergence delirium, *IL-6* Interleukin-6, *MMP-9* Metalloproteinase-9, *ND* Not descripted, *NA* Not applicable, *POCD* Postoperative cognitive dysfunction, *PCCs* Perioperative cognitive complications, *PACU* post-anesthesia care unit, *CGRP* Calcitonin gene-related peptide, *MMSE* Mini-mental State Examination, *RASS* Richmond Agitation-Sedation Scale, *CAM* The Confusion Assessment Method

### Primary endpoint

**Fig. 2:**
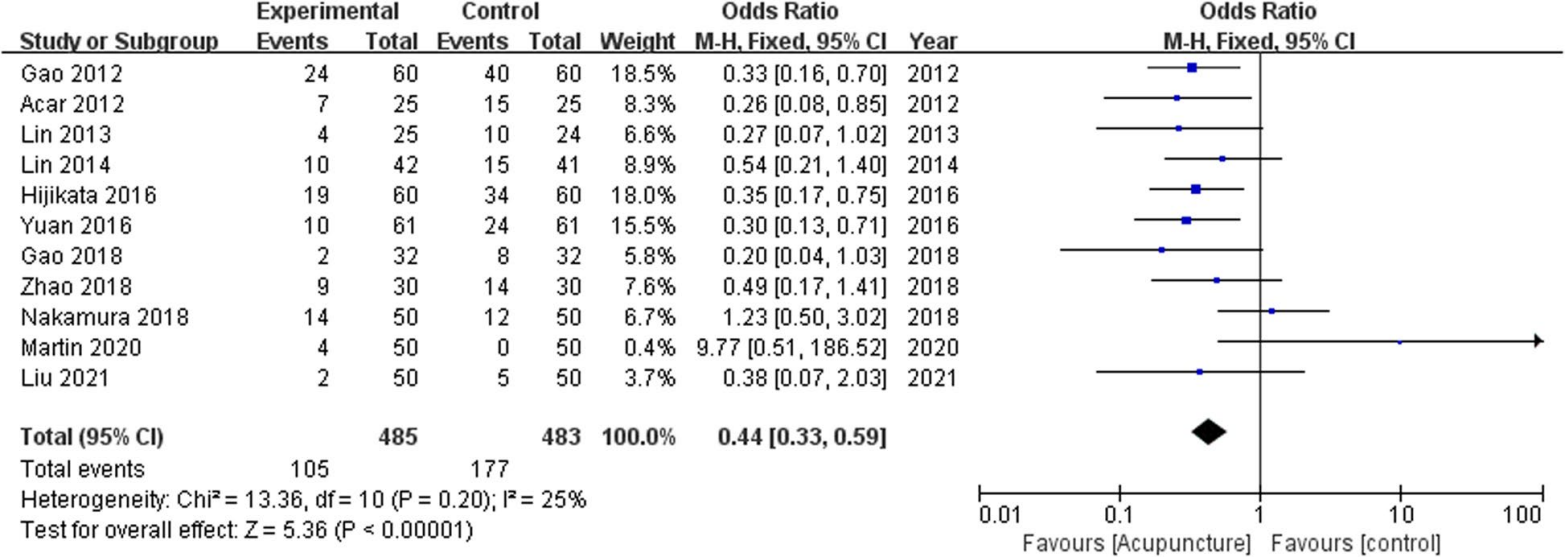
Forest plot for the incidence of PCC during hospital stay. The plot shows decreased incidence in patients treated with acupuncture-related techniques compared with non-acupuncture controls. Fixed-effects odds ratios are calculated using the Mantel–Haenszel test. Error bars represent 95% CI. OR = odds ratio

In one study (Zhang et al. 2017), only highest MMSE scores were recorded and the incidence of PPC could not be determined. Eleven studies comprising 968 participants provided data on the incidence of postoperative cognitive complications. A low certainty of evidence supported that perioperative administration of acupuncture-related techniques was associated with a lower incidence of postoperative cognitive complications (OR, 0.44; 95% CI, 0.33 to 0.59; *P* < 0.00001; df = 10; *I*^2^ = 26%; Fig. 2 and Additional file 1: Table S2). Egger’s regression test for assessing publication bias showed no significant bias (*P* = 0.27).

### Postoperative delirium and delayed cognitive recovery

Five trials comprising 490 participants assessed postoperative delirium. Acupuncture was associated with a lower incidence of postoperative delirium (OR, 0.51; 95% CI, 0.34 to 0.76; *P* < 0.001; df = 4; *I*^2^ = 64%; Additional file 1: Fig. S2). Seven trials comprising 478 participants evaluated delayed cognitive recovery. Acupuncture was associated with a lower incidence of delayed cognitive recovery (OR, 0.33; 95% CI, 0.21 to 0.51; *P* < 0.001; df = 5; *I*^2^ = 0%; Additional file 1: Fig. S2).

### Acupuncture with needle and acupuncture without needle

Three trials comprising 243 participants compared acupuncture with needles and control conditions. The incidence of postoperative cognitive disorder was not different between the two techniques (OR, 0.56; 95% CI, 0.30 to 1.05; *P* = 0.07; df = 2; *I*^2^ = 59%; Additional file 1: Fig. S3). Eight trials comprising 725 participants compared acupuncture without needles and control conditions. Acupuncture was associated with a lower incidence of postoperative cognitive disorder (OR, 0.38; 95% CI, 0.27 to 0.54; *P* < 0.001; df = 7; *I*^2^ = 16%; Additional file 1: Fig. S3).

### Pediatric patients and non-pediatric patients

Subgroup analysis showed that based on the four trials comprising 370 participants compared acupuncture and control conditions in pediatric patients, acupuncture was associated with a lower incidence of postoperative cognitive complications (OR, 0.61; 95% CI, 0.38 to 0.98; *P* = 0.04; df = 3; *I*^2^ = 69%; Additional file 1: Fig. S4). Seven trials comprising 598 participants compared acupuncture and control conditions in non-pediatric patients. Acupuncture was associated with a lower incidence of postoperative cognitive complications (OR, 0.36; 95% CI, 0.24 to 0.53; *P* < 0.001; df = 6; *I*^2^ = 0%; Additional file 1: Fig. S4).

### Secondary end points

**Fig. 3:**
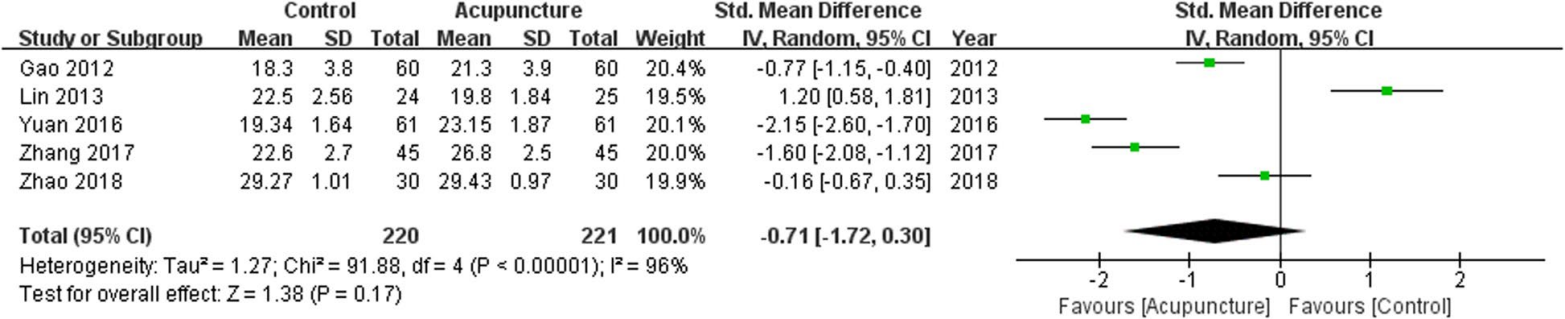
Forest plot for the highest MMSE scores during hospital stay. The plot shows that MMSE scores were not increased in patients treated with acupuncture-related techniques compared with non-acupuncture controls. Standard mean differences (SMD) are calculated. MMSE = Mini-mental status examination

Five trials in adult patients only comprising 441 participants compared the highest MMSE scores obtained during the hospital stay. No difference was observed between participants treated with acupuncture and those in the control group (SMD, − 0.71; 95% CI, − 1.72 to 0.30; *P* = 0.17; df = 4; *I*^2^ = 96%; Fig. 3).

**Fig. 4:**
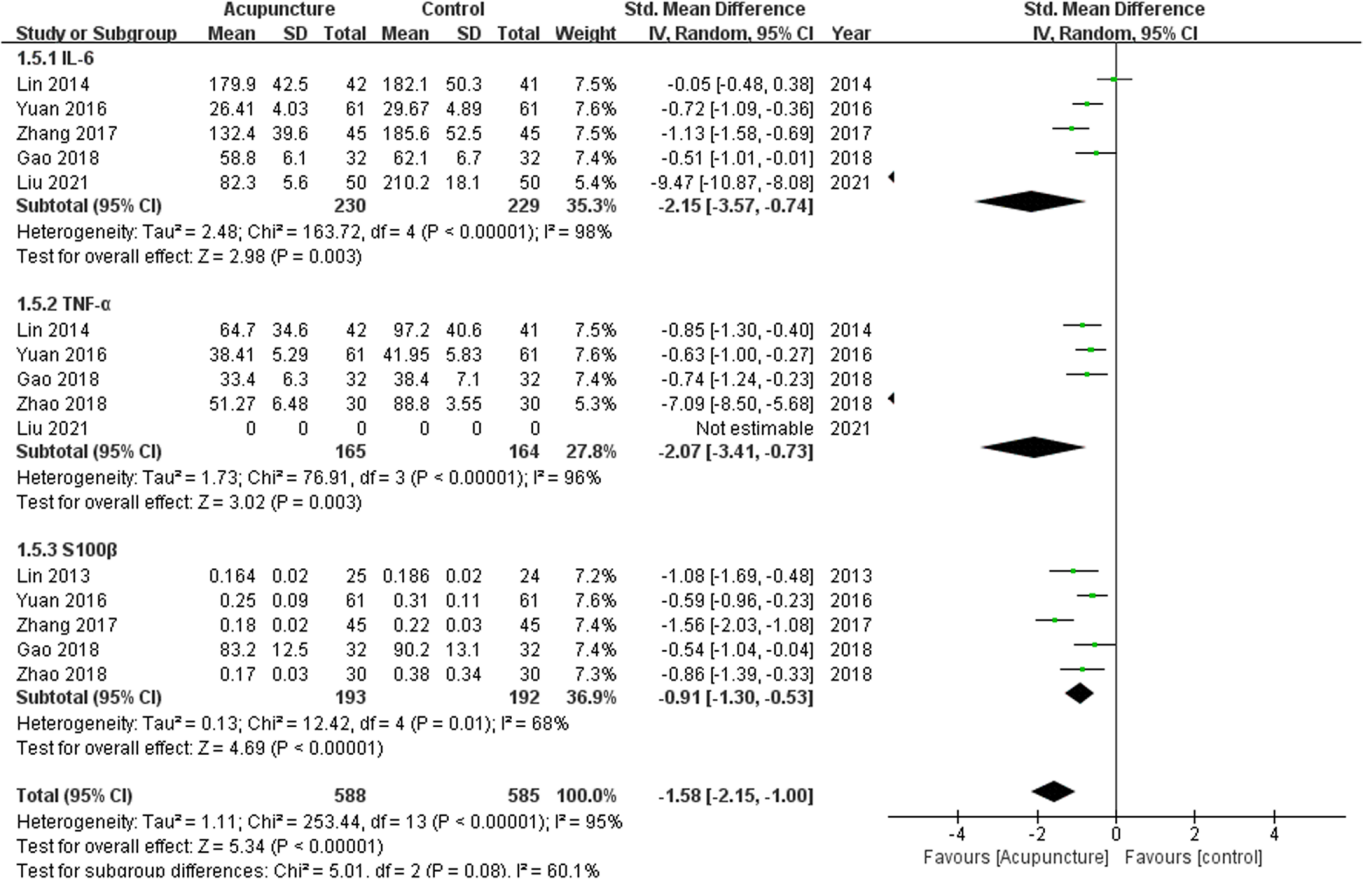
Forest plot for the highest level of serum inflammatory cytokines and brain injury markers during hospital stay. The plot shows decreased level of markers in patients treated with acupuncture-related techniques compared with non-acupuncture controls. Standard mean differences (SMD) are calculated

Five trials comprising 459 participants compared serum IL-6 levels. Acupuncture was associated with a lower level of IL-6 (SMD, − 2.51; 95% CI, − 3.57 to − 0.74; *P* = 0.003; df = 4; *I*^2^ = 98%; Fig. 4). Five trials comprising 329 participants and 385 participants compared the levels of serum TNF-α and S100β, respectively. Acupuncture was associated with lower levels of TNF-α (SMD, − 2.07; 95% CI, − 3.41 to − 0.73; *P* = 0.003; df = 4; *I*^2^ = 96%) and S100β (SMD, − 0.91; 95% CI, − 1.3 to − 0.53; *P* < 0.00001; df = 4; *I*^2^ = 68%; Fig. 4). The certainty of the evidence was very low (Additional file 1: Table S2).

### Sensitivity analysis

In one trial reporting the incidence of postoperative cognitive complications, the number of events in the control group was zero. For sensitivity analyses with continuity corrections, 1 was added to each cell of the 2 × 2 tables from the trial. Acupuncture was associated with a lower incidence of postoperative cognitive complications (OR, 0.44; 95% CI, 0.33 to 0.60; *P* < 0.00001; df = 10; *I*^2^ = 30%; Additional file 1: Fig. S5).

Seven trials were published in English, and four trials were published in non-English (all Chinese). The results of trials published in English (*n* = 594; OR, 0.52; 95% CI, 0.35 to 0.79, *P* = 0.002) and non-English (*n* = 394; OR, 0.35; 95% CI, 0.22 to 0.55, *P* < 0.00001) both showed significantly fewer postoperative cognitive complications in patients who received the acupuncture-related interventions (Additional file 1: Fig. S6).

Six trials used propofol, and five trials used sevoflurane for general anesthesia maintenance. Acupuncture-related techniques were associated with significantly lower rates of PCCs regardless of the type of anesthetics used for general anesthesia (both *P* < 0.001, Additional file 1: Fig. S7).

## Discussion

Our review of acupuncture-related techniques as interventions for postoperative cognitive complications found low-certainty evidence that showed that acupuncture-related techniques, compared with control conditions, were associated with reduced postoperative cognitive complications. Subgroup analyses reported that both acute agitation and/or delirium and delayed cognitive recovery were lower in participants who received acupuncture-related techniques. The incidence of postoperative cognitive complications was lower in participants received acupuncture in both pediatric population and non-pediatric population.

In this study, we used the mesh term ‘postoperative cognitive complications’. Another nomenclature 'perioperative neurocognitive disorders’ was also recommended to be used as an overarching term for cognitive impairment identified in the preoperative or postoperative period (Evered et al. 2018). It includes any form of acute event (postoperative delirium) and cognitive decline diagnosed up to 30 days (delayed neurocognitive recovery) and up to 12 months (postoperative neurocognitive disorder) after the procedure. Based on this definition, the cognitive changes after surgery assessed in the included studies were acute delirium and delayed neurocognitive recovery. In the studies included, MMSE was only undertaken as the primary outcome diagnostic tool in patients older than 65 years The MMSE is reported as the diagnostic tool for agitation and delirium in adults only, adding evidence that there are different pathologies presenting as agitations and/or delirium in older adults and in children. As indicated by the subgroup analysis, in pediatric patients, acupuncture was associated with a lower incidence of agitation and/or delirium, which is in accordance with the less cognitive complications in adults.

Our review found very low-certainty evidence that MMSE scores were higher and the levels of proinflammatory biomarkers, including serum IL-6, TNF-α and S100β, were lower in patients treated with acupuncture. This concurs with a previous report that postoperative cognitive dysfunction is correlated with the concentrations of peripheral inflammatory markers, particularly interleukin-6 and S-100β (Wiberg et al. 2021; Peng et al. 2013). The role of inflammation in perioperative brain function is becoming apparent (Subramaniyan and Terrando 2019). Surgery may induce a systemic inflammatory response via cytokines such as IL-1β (Cibelli et al. 2010), TNF-α (Terrando et al. 2010), and IL-6 (Hu et al. 2018), as well as S100 Ca^2+^-binding proteins and oxidative stress pathways. Administration of an IL-6 monoclonal antibody and targeting of TNF-α have been reported to prevent postoperative cognitive complications (Cibelli et al. 2010; Hu et al. 2018; Subramaniyan and Terrando 2019; Terrando et al. 2010). There is a potential benefit of acupuncture in terms of inflammatory markers and it requires further investigation.

Regardless of the type of acupuncture technique, the effect of decreasing postoperative cognitive complications could be induced by manual or electrical stimulation (transcutaneous electrical acupoint stimulation, TEAS). The frequency of the stimulation regimen for electroacupuncture was low (1 Hz, 2 Hz, 10 Hz, 15 Hz). Various studies have reported that high-frequency and low-frequency electroacupuncture or TEAS have clinical effects elicit through distinct mechanisms (Yang et al. 2016). For example, the suppressive effects of low-frequency electroacupuncture on carrageenan-induced edema and pain are mediated by sympathetic postganglionic neurons, while the suppressive effects of HF EA are mediated by the sympatho-adrenal medullary axis (Kim et al. 2008). Moreover, an animal study showed that high-frequency electroacupuncture (50 Hz) more effectively exerted a protective effect against Aβ_1-42_-induced learning and memory deficits and synapse ultrastructure impairments (Yu et al. 2018). The most effective electroacupuncture frequency for postoperative cognitive complications remains to be explored. The comparators were no acupuncture, inactive plaster or no stimulation electrode control (which is called mock TEAS). It was recommended that regardless of the choice of control group, it is valuable to check its adequacy (Vincent and Lewith 1995). Nevertheless, no information about verifying the adequacy of the control conditions was provided in the studies included in our review. The timing of acupuncture varies in enrolled studies. Acupuncture was given before the surgery in eight studies and during the surgery in four studies. It was speculated that acupuncture provided after anesthesia induction may not be as effective as that provided when patients were awake (Yang et al. 2016). Further studies were needed to verify the optimal timing of perioperative acupuncture.

The trials on pediatric patients were mostly performed in countries outside China, and the measured outcomes were agitation and/or delirium, which is likely an emergence delirium. The trials on non-pediatric patients were mostly on patients older than 65 years old and focused on delayed cognitive recovery. The number of acupoints chosen across interventions varied greatly, and a comparison of the effect of acupuncturing different acupoints was difficult. Baihui, Hegu, and Neiguan were the most frequently used acupoints (Baihui in 6 trials, Hegu in 4 trials, and Neiguan in 6 trials). The timing of acupuncture included preoperative, intraoperative and postoperative and a combination of the three treatment times. Seven trials administered TEAS 30 min before anesthesia induction, which is a frequently used protocol for many studies on perioperative acupuncture (Lu et al. 2021).

Our review could not adequately assess adverse events associated with acupuncture-related techniques. All studies included in our review were published after the release of the Consolidated Standards of Reporting Trials (CONSORT) statement in 1996 (Begg et al. 1996), which recommended that trial investigators report unintended effects related to interventions. However, no study provided information of adverse events. Whether acupuncture-related techniques are related to significant adverse events still needs further investigation.

The strengths of our study include the comprehensive nature of the literature search, which identified 12 publications, thus permitting sensitivity analyses. In addition, we complied with the Cochrane methodology. We attempted to contact the authors to more accurately evaluate the risk of bias and to obtain necessary unpublished data. We also confirmed the robustness of our findings with sensitivity analysis.

Our review also has limitations. First, age ranges of the participants in the enrolled trials were different. Second, the JADAD score of the eligible studies was relatively low. Seven trials had a JADAD score of 2, and one trial scored 1 (Additional file 1: Table S1). The assessment of evidence grade was low and very low for the measured outcomes (Additional file 1: Table S2). Risk of bias and incomplete reporting were major concerns contributing to the low quality of the evidence. Risk of bias resulted from difficulty in blinding for the acupuncture intervention, though it could be partly overcome by blinded outcome assessment Incomplete reporting was based on the failure to provide values of some effects, including the 95% CI, in some trials. There was a risk of multiple testing bias in our secondary outcomes, as a 95% CI was calculated. In relation to this, methodological bias continues to be a concern, as some trials were characterized by an unclear risk of bias. Third, the surgery type varies in the enrolled studies. Data retrieved from same surgeries may provide more potent evidence. Moreover, no data were available on postoperative cognitive disturbance-related outcomes beyond 30 days after surgery. Whether acupuncture like other non-pharmacological interventions have no effect on long-term cognitive disturbance reported after delirium needs to be verified.

## Conclusions

This systemic review and meta-analysis reports that acupuncture-related techniques may decrease post-operative cognitive complication and warrants further investigation. Nevertheless, the inherent limitations of the included studies prevent us from reaching definitive conclusions. Future large, well-design ed RCTs with extensive follow-up are needed to confirm and update the findings of this analysis.

## Supplementary Information

**Additional file 1: Supplemental file 1 (eMethod).** Search Strategy. **Table S1.** Study Quality of Eligible Trials. **Table S2.** Grading of Recommendations Assessment, Development and Evaluation Summary of Quality of Evidence for primary and secondary Outcomes. **Figure S1.** Risk of bias summary: review authors' judgements about each risk of bias item for each included study. **Figure S2.** Forest plot for subgroup analysis of the incidence of PND by type of PND. **Figure S3.** Forest plot for subgroup analysis of the incidence of PND by acupuncture techniques. **Figure S4.** Forest plot for subgroup analysis of the incidence of PND by age. **Figure S5.** Sensitivity analyses with continuity corrections on zero events of PND incidence. **Figure S6.** Effect of acupuncture on PND in studies published in English and non-English. **Figure S7.** Effect of acupuncture on PND under different anesthetic techniques. **Figure S8.** Funnel Plots for Studies Evaluating.

## Data Availability

All data generated or analyzed during this study are included in this published article, its supplementary information files, and the primary randomized controlled trials cited for inclusion. A copy of the raw data could be reached by requests for additional analysis. Data will be available when the manuscript published.

## Abbreviations

PCC: Postoperative cognitive complications
PRISMA: Preferred Reporting Items for Systematic Reviews and Meta-Analyses
MMSE: Mini-mental state examination
IL: Interleukin
TNF: Tumor necrosis factor
ASA: American Society of Anesthesiologists
GRADE: Grading of Recommendations Assessment, Development and Evaluation
OR: Odds ratio
SMD: Standardized mean difference
CI: Confidence interval
RCT: Randomized clinical trial
ENT: Ear, nose and throat
TEAS: Transcutaneous electrical acupoint stimulation
CONSORT: Consolidated Standards of Reporting Trials
