# Supplementary material for: Acupuncture-related techniques for postoperative cognitive complications: a systemic review and meta-analysis
Source: Perioper Med (Lond). 2023 May 3;12:14. doi: 10.1186/s13741-023-00303-5 (PMC10155419; doi:10.1186/s13741-023-00303-5)
Supplement: Supplementary file 1 — Additional file 1: Supplemental file 1 (eMethod). Search Strategy. Table S1. Study Quality of Eligible Trials. Table S2. Grading of Recommendations Assessment, Development and Evaluation Summary of Quality of Evidence for primary and secondary Outcomes. Figure S1. Risk of bias summary: review authors' judgements about each risk of bias item for each included study. Figure S2. Forest plot for subgroup analysis of the incidence of PND by type of PND. Figure S3. Forest plot for subgroup analysis of the incidence of PND by acupuncture techniques. Figure S4. Forest plot for subgroup analysis of the incidence of PND by age. Figure S5. Sensitivity analyses with continuity corrections on zero events of PND incidence. Figure S6. Effect of acupuncture on PND in studies published in English and non-English. Figure S7. Effect of acupuncture on PND under different anesthetic techniques. Figure S8. Funnel Plots for Studies Evaluating. [file 13741_2023_303_MOESM1_ESM.docx]

**Supplementary Online Content**

Supplemental file 1(eMethod). Search Strategy

Supplemental file 2 (eTable 1). Study Quality of Eligible Trials

Supplemental file 3. (eTable 2) Grading of Recommendations Assessment, Development and Evaluation Summary of Quality of Evidence for primary and secondary Outcomes

Supplemental file 4 (eFigure 1) Risk of bias summary: review authors' judgements about each risk of bias item for each included study

Supplemental file 5 (eFigure 2) Forest plot for subgroup analysis of the incidence of PND by type of PND

Supplemental file 6 (eFigure 3) Forest plot for subgroup analysis of the incidence of PND by acupuncture techniques

Supplemental file 7 (eFigure 4) Forest plot for subgroup analysis of the incidence of PND by age

Supplemental file 8 (eFigure 5) Sensitivity analyses with continuity corrections on zero events of PND incidence

Supplemental file 9 (eFigure 6). Effect of acupuncture on PND in studies published in English and non-English

Supplemental file 10 (eFigure 7). Effect of acupuncture on PND under different anesthetic techniques

Supplemental file 11(eFigure 8). Funnel Plots for Studies Evaluating

eMethods. Search Strategy

**EMBase**

| **No.** | **Query** | **Results** |
| --- | --- | --- |
| #1 | acupuncture:ti,ab,kw OR electroacupuncture:ti,ab,kw OR 'transcutaneous electrical acupoint stimulation':ti,ab,kw OR 'acupuncture, ear':ti,ab,kw OR 'laser acupuncture':ti,ab,kw OR acupressure:ti,ab,kw | 38319 |
| #2 | ‘emergence delirium':ti,ab,kw OR ‘emergence agitation':ti,ab,kw OR 'postoperative cognitive dysfunction':ti,ab,kw OR 'postoperative cognitive decline':ti,ab,kw OR 'postoperative cognitive complication':ti,ab,kw OR 'postoperative delirium':ti,ab,kw | 5137 |
| #3 | #1 AND #2 | 32 |

**PubMed**

| **No.** | **Query** | **Results** |
| --- | --- | --- |
| #1 | "acupuncture"[MeSH Terms] OR "acupuncture therapy"[MeSH Terms] OR "electroacupuncture"[MeSH Terms] OR "transcutaneous electrical acupoint stimulation"[Title/Abstract] OR "transcutaneous electrical acustimulation"[Title/Abstract] OR "acupuncture, ear"[MeSH Terms] OR "acupuncture, ear"[MeSH Terms] OR "laser acupuncture"[Title/Abstract] OR "acupressure"[MeSH Terms] OR "acupoint stimulation"[Title/Abstract] | 27225 |
| #2 | "postoperative cognitive complications"[MeSH Terms] OR "emergence delirium"[MeSH Terms] OR "postoperative cognitive dysfunction"[Title/Abstract] OR "postoperative cognitive decline"[Title/Abstract] OR "postoperative delirium"[Title/Abstract] OR "perioperative neurocognitive disorder"[Title/Abstract] OR "emergence agitation"[Title/Abstract] | 3724 |
| #3 | #1 AND #2 | 24 |

**Web of Science**

| **No.** | **Query** | **Results** |
| --- | --- | --- |
| #1 | TS=("postoperative cognitive complications"OR "emergence delirium") OR AB=("postoperative cognitive dysfunction" OR "postoperative cognitive decline" OR "postoperative delirium" OR "perioperative neurocognitive disorder" OR "emergence agitation")  *Indexes=SCI-EXPANDED, SSCI, A&HCI, ESCI Timespan=All years* | 3094 |
| #2 | TS=("acupuncture" OR "acupuncture therapy" OR "electroacupuncture" OR "acupuncture, ear" OR "acupuncture, ear" OR "acupressure") OR AB=( "transcutaneous electrical acupoint stimulation" OR "transcutaneous electrical acustimulation"OR "laser acupuncture" OR "acupoint stimulation")  *Indexes=SCI-EXPANDED, SSCI, A&HCI, ESCI Timespan=All years* | 22935 |
| #3 | #2 AND #1  *Indexes=SCI-EXPANDED, SSCI, A&HCI, ESCI Timespan=All years* | 26 |

**Cochrane database**

| **No.** | **Query** | **Results** |
| --- | --- | --- |
| #1 | MeSH descriptor: [Acupuncture] explode all trees | 154 |
| #2 | MeSH descriptor: [Acupressure] explode all trees | 377 |
| #3 | MeSH descriptor: [Acupuncture therapy] explode all trees | 4933 |
| #4 | MeSH descriptor: [Acupuncture, ear] explode all trees | 199 |
| #5 | MeSH descriptor: [Electrocupuncture] explode all trees | 837 |
| #6 | (transcutaneous electrical acupoint stimulation):ti,ab,kw OR (transcutaneous electrical acustimulation):ti,ab,kw OR (laser acupuncture):ti,ab,kw OR (acupoint stimulation):ti,ab,kw | 1280 |
| #7 | #1 OR #2 OR #3 OR #4 OR #5 OR #6 | 6126 |
| #8 | MeSH descriptor: [Postopertive Cognitive Complicaitons] explode all trees | 21 |
| #9 | MeSH descriptor: [Emergence Delirium] explode all trees | 108 |
| #10 | (emergence agitation):ti,ab,kw OR (postoperative cognitive dysfunction):ti,ab,kw OR (postoperative cognitive decline):ti,ab,kw OR (postoperative delirium):ti,ab,kw | 2870 |
| #11 | #8 OR #9 OR #10 | 2888 |
| #12 | #7 AND #11 | 24 |

**ClinicalTrials.gov**

Condition or disease: postoperative cognitive dysfunction; postoperative cognitive decline; postoperative delirium

Other terms: acupuncture; transcutaneous electrical acupoint stimulation; electroacupuncture; acupressure; laser acupuncture

Rsults=8

**eTable 1. Study Quality of Eligible Trials**

| **Study** | **Year** | **Type of study** | **Type of PND** | **Study described as randomized?** | **Randomization method described and appropriate?** | **Study described as double blind?** | **Method of double blinding described and appropriate?** | **Description of withdrawals and dropouts?** | **Jadad Score (0-5)** |
| --- | --- | --- | --- | --- | --- | --- | --- | --- | --- |
| Lin^[1]^ | 2009 | RCT | Emergence agitation | Yes | NA | NA | NA | NA | 1 |
| Acar^[2]^ | 2012 | RCT | Emergence agitation | Yes | Yes | Yes | Yes | NA | 4 |
| Gao^[3]^ | 2012 | RCT | POCD | Yes | Yes | NA | NA | NA | 2 |
| Lin^[4]^ | 2013 | RCT | POCD | Yes | Yes | NA | NA | NA | 2 |
| Lin^[5]^ | 2014 | RCT | POCD | Yes | Yes | NA | NA | NA | 2 |
| Hijikata^[6]^ | 2016 | RCT | Emergence agitation | Yes | Yes | Yes | Yes | NA | 4 |
| Yuan^[7]^ | 2016 | RCT | POCD | Yes | Yes | NA | NA | NA | 2 |
| Zhang^[8]^ | 2017 | RCT | POCD | Yes | Yes | NA | NA | NA | 2 |
| Gao^[9]^ | 2018 | RCT | POD | Yes | Yes | NA | NA | NA | 2 |
| Nakamura^[10]^ | 2018 | RCT | Emergence delirium | Yes | Yes | Yes | Yes | NA | 4 |
| Zhao^[11]^ | 2018 | RCT | POCD | Yes | Yes | Yes | Yes | Yes | 5 |
| Martin^[12]^ | 2020 | RCT | Emergence delirium | Yes | Yes | Yes | Yes | NA | 4 |
| Liu^[13]^ | 2020 | RCT-Pilot study | POCD | Yes | Yes | NA | NA | NA | 2 |

RCT: Randomized clinical trial; PND: Perioperative neurocognitive disorder; POCD: Postoperative cognitive dysfunction; POD: Postoperative delirium; NA: Not applicable

**eTable 2. Grading of Recommendations Assessment, Development and Evaluation Summary of Quality of Evidence for primary and secondary Outcomes**

| **Outcomes** | **Illustrative comparative risks* (95% CI)** | | **Relative effect**  **(95% CI)** | **No of Participants**  **(studies)** | **Quality of the evidence**  **(GRADE)** | **Comments** |
| --- | --- | --- | --- | --- | --- | --- |
|  | Assumed risk | Corresponding risk |  |  |  |  |
|  | Control | PND |  |  |  |  |
| Incidence of PND  OR | Study population | | OR 0.44  (0.33 to 0.59) | 968  (11 studies) | ⊕⊕⊝⊝  low^1,2^ |  |
|  | 366 per 1000 | 203 per 1000  (160 to 254) |  |  |  |  |
|  | Moderate | |  |  |  |  |
|  | 393 per 1000 | 222 per 1000  (176 to 276) |  |  |  |  |
| MMSE score  SMD |  | The mean MMSE score in the intervention groups was 0.9 standard deviations lower  (1.1 to 0.69 lower) |  | 441 (5 studies) | ⊕⊝⊝⊝  very low^1,3^ | SMD -0.9 (-1.1 to -0.69) |
| IL-6  SMD |  | The mean IL-6 in the intervention groups was 0.81 standard deviations lower  (1.03 to 0.6 lower) |  | 459 (5 studies) | ⊕⊝⊝⊝  very low^1,3^ | SMD -0.81 (-1.03 to -0.6) |
| TNF-α  SMD |  | The mean TNF-α in the intervention groups was 0.91 standard deviations lower  (1.16 to 0.67 lower) |  | 329 (4 studies) | ⊕⊝⊝⊝ very low^1,3^ | SMD -0.91 (-1.16 to -0.67) |
| S100β  SMD |  | The mean S100β in the intervention groups was 0.88 standard deviations lower  (1.09 to 0.67 lower) |  | 385 (5 studies) | ⊕⊝⊝⊝  very low^1,3^ | SMD -0.88 (-1.09 to -0.67) |

*The basis for the assumed risk (e.g. the median control group risk across studies) is provided in footnotes. The corresponding risk (and its 95% confidence interval) is based on the assumed risk in the comparison group and the relative effect of the intervention (and its 95% CI). CI: Confidence interval; OR: Odds ratio; SMD: standard mean difference

^1^ Blinding was not possible due to the characteristic of the intervention

^2^ No 95%CI could be calculated for 3 of the trials

^3^ No 95% CI could be calculated for most of the trials


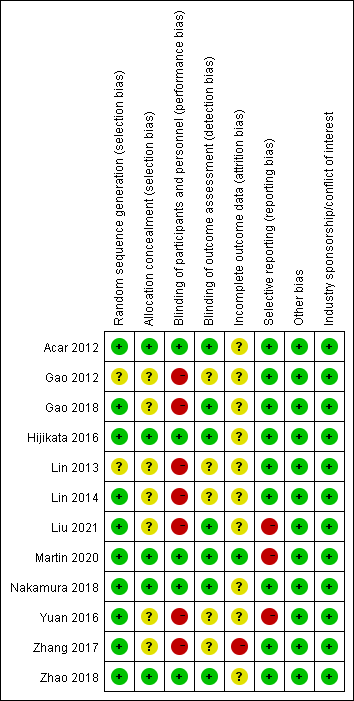


**eFigure 1. Risk of bias summary: review authors' judgements about each risk of bias item for each included study**


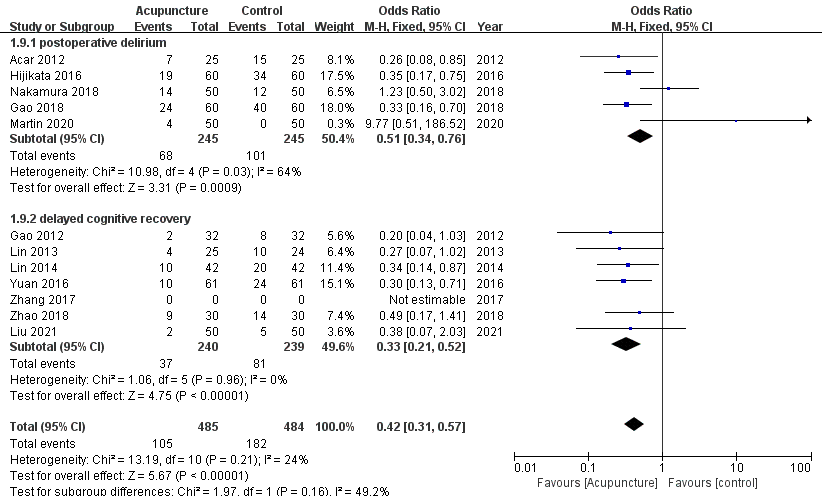


**eFigure 2. Forest plot for subgroup analysis of the incidence of PND by type of PND.**

The plot shows decreased incidence of both delirium and delayed cognitive recovery in patients treated with acupuncture related techniques compared with non-acupuncture controls. Fixed-effects odds ratios are calculated using the Mantel-Haenszel test. Error bars represent 95% CI. OR = Odds ratio


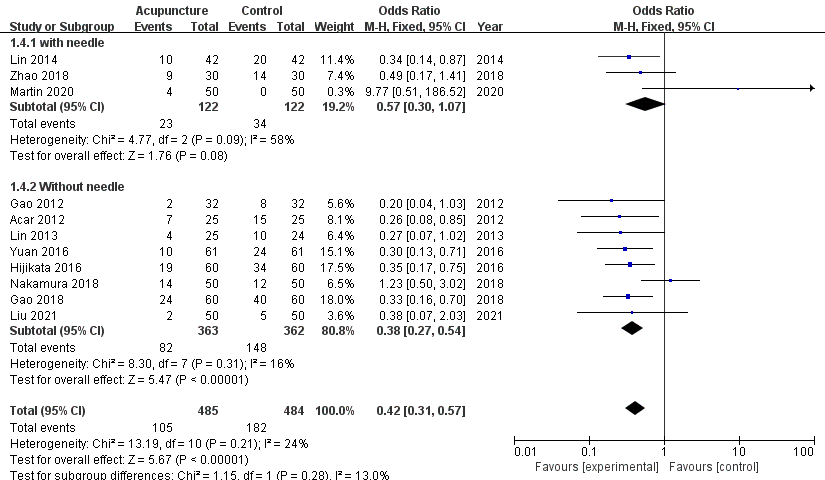


**eFigure 3. Forest plot for subgroup analysis of the incidence of PND by acupuncture techniques.**

The plot shows decreased incidence of PND in patients treated with all kinds of acupuncture related techniques compared with non-acupuncture controls. Fixed-effects odds ratios are calculated using the Mantel-Haenszel test. Error bars represent 95% CI. OR = Odds ratio


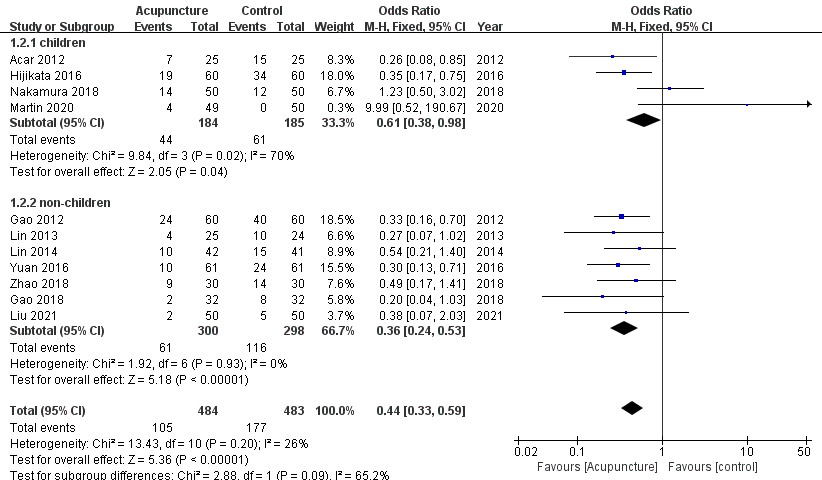


**eFigure 4. Forest plot for subgroup analysis of the incidence of PND by age.**

The plot shows decreased incidence of PND in both pediatric patients and adult patients treated with acupuncture related techniques compared with non-acupuncture controls. Fixed-effects odds ratios are calculated using the Mantel-Haenszel test. Error bars represent 95% CI. OR = Odds ratio


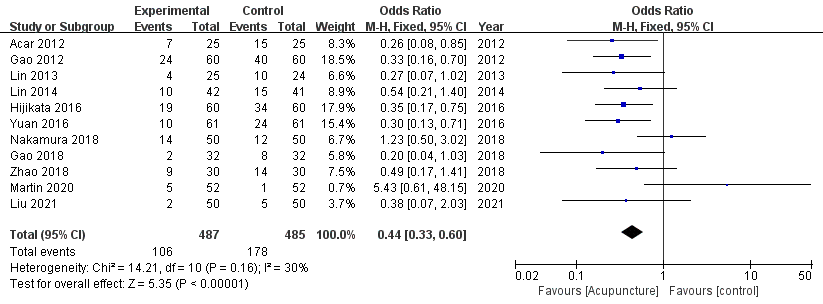


**eFigure 5 Sensitivity analyses with continuity corrections on zero events of PND incidence**


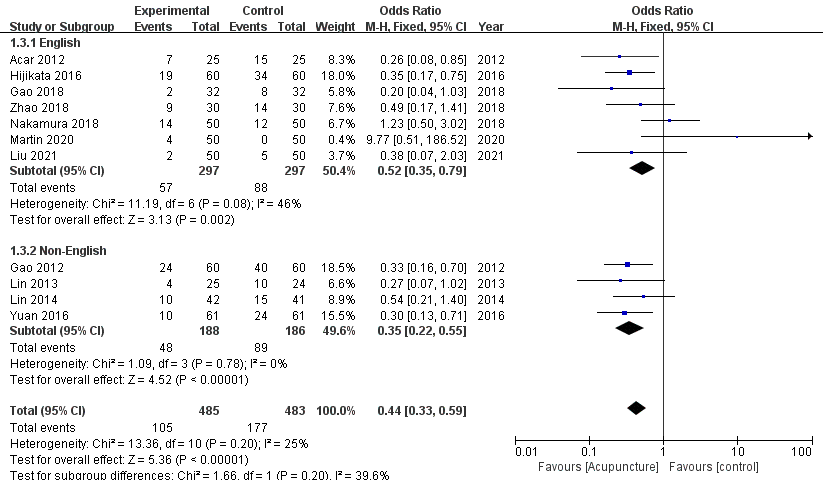


**eFigure 6. Incidence of PND in trials published in English and non-English.**


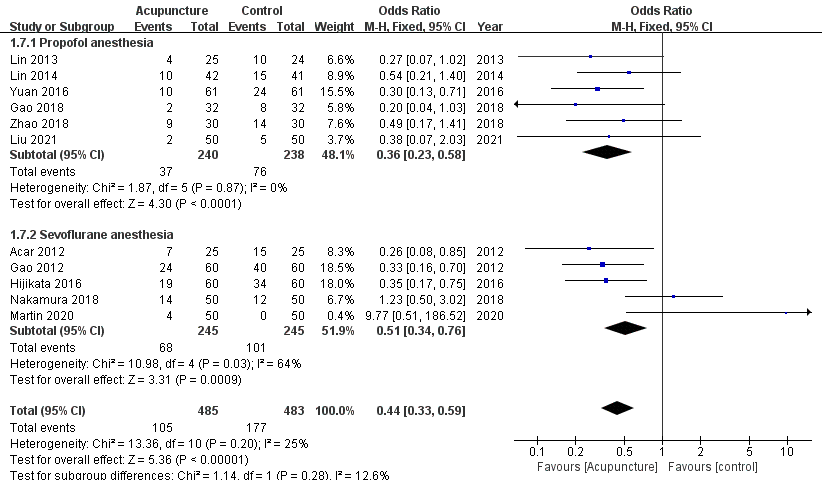


**eFigure 7. Incidence of PND in trials using propofol anesthesia and sevoflurane anesthesia.**


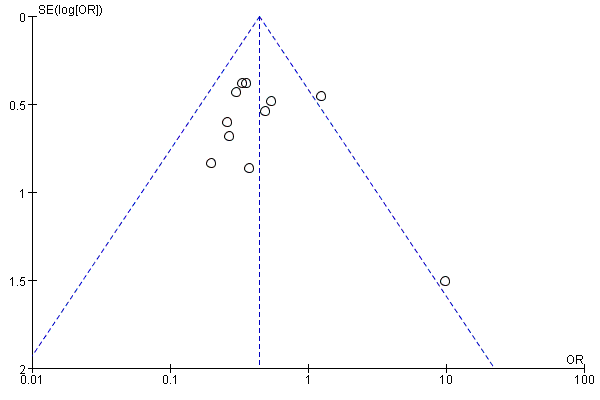


**eFigure 8. Funnel Plots for Studies Evaluating**

**References:**

1. Lin YC, Tassone RF, Jahng S, et al. Acupuncture management of pain and emergence agitation in children after bilateral myringotomy and tympanostomy tube insertion*.* *Paediatr Anaesth*. 2009; 19(11): 1096-101.

2. Acar HV, Yilmaz A, Demir G, et al. Capsicum plasters on acupoints decrease the incidence of emergence agitation in pediatric patients*.* *Pediatric Anesthesia*. 2012; 22(11): 1105-1109.

3. Gao XQ, Zhang ZY, and Ma WH. [Effects of electroacupuncture assistant general anesthesia on postoperative cognitive dysfunction of aged patients]*.* *Chinese journal of integrated traditional and Western medicine (Zhongguo Zhong Xi Yi Jie He Za Zhi)*. 2012; 32(5): 591-593.

4. Lin SY, Gao J, Yin ZL, et al. Impacts of the different frequencies of electroacupunctrue on cognitive function in patients after abdominal operation under compound anesthesia of acupuncture and drugs*.* *Zhongguo zhen jiu [Chinese acupuncture & moxibustion]*. 2013; 33(12): 1109‐1112.

5. Lin SY, Yin ZL, Gao J, et al. [Effect of acupuncture-anesthetic composite anesthesia on the incidence of POCD and TNF-alpha, IL-1beta, IL-6 in elderly patients]*.* *Chinese journal of integrated traditional and Western medicine (Zhongguo Zhong Xi Yi Jie He Za Zhi)*. 2014; 34(7): 795-9.

6. Hijikata T, Mihara T, Nakamura N, et al. Electrical stimulation of the heart 7 acupuncture site for preventing emergence agitation in children: A randomised controlled trial*.* *Eur J Anaesthesiol*. 2016; 33(7): 535-42.

7. Yuan J, Wu Y, Li JY, et al. [Effect of Dexmedetomidine Combined Electrical Stimulation on Coanitive Function of Patients Receiving Extracerebral Intervention]*.* *Chinese journal of integrated traditional and Western medicine (Zhongguo Zhong Xi Yi Jie He Za Zhi)*. 2016; 36(3): 285-8.

8. Zhang Q, Li YN, Guo YY, et al. Effects of preconditioning of electro-acupuncture on postoperative cognitive dysfunction in elderly: A prospective, randomized, controlled trial*.* *Medicine (Baltimore)*. 2017; 96(26): e7375.

9. Gao F, Zhang Q, Li Y, et al. Transcutaneous electrical acupoint stimulation for prevention of postoperative delirium in geriatric patients with silent lacunar infarction: a preliminary study*.* *Clin Interv Aging*. 2018; 13: 2127-2134.

10. Nakamura N, Mihara T, Hijikata T, et al. Unilateral electrical stimulation of the heart 7 acupuncture point to prevent emergence agitation in children: A prospective, double-blinded, randomized clinical trial*.* *PLoS One*. 2018; 13(10): e0204533.

11. Zhao FY, Zhang ZY, Zhao YX, et al. The effect of electroacupuncture preconditioning on cognitive impairments following knee replacement among elderly: A randomized controlled trial*.* *World Journal of Acupuncture-Moxibustion*. 2018; 28(4): 231-236.

12. Martin CS, Yanez ND, Treggiari MM, et al. Randomized controlled trial of acupuncture to prevent emergence delirium in children undergoing myringotomy tube placement*.* *Minerva Anestesiologica*. 2020; 86(2): 141-149.

13. Liu T, Yin C, Li Y, et al. Effects of Transcutaneous Electrical Acupoint Stimulation on Postoperative Cognitive Decline in Elderly Patients: A Pilot Study*.* *Clin Interv Aging*. 2021; 16: 757-765.
